# New Medical School

**Published:** 1859-05

**Authors:** 


New Medical School.—A faculty has been recently organized at Mobile, Alabama, for the founding of a medical college. Dr. J. C. Nott has the Chair of Surgery, and will sail for Europe early in May, for the purpose of purchasing a museum and apparatus for the institution.

